# Evolution of Holes and Cracks in Pre-Carbonized Glassy Carbon

**DOI:** 10.3390/ma17215274

**Published:** 2024-10-30

**Authors:** Yi Yang, Wei Wang, Haihui Ruan

**Affiliations:** Department of Mechanical Engineering, The Hong Kong Polytechnic University, Hung Hom, Hong Kong; 20099079r@connect.polyu.hk (Y.Y.); 21040463r@connect.polyu.hk (W.W.)

**Keywords:** pre-carbonized glassy carbon, phenol formaldehyde resin, pyrolysis, characterization, molding application

## Abstract

Being a type of carbonaceous material, glassy carbon possesses thermomechanical properties akin to ceramics, offering both mechanical and chemical stability at high temperatures; therefore, it can be applied in electrochemistry and high-temperature manufacturing. However, the direct pyrolysis of a bulk precursor leads to internal pores and cracks, usually resulting in fracture. Our characterization results show that at temperatures below 400 °C, large pores do not form, and pre-carbonized glassy carbon (PGC) formed at 350 °C has a dense microstructure without cracks. It exhibits a high compressive strength of ~370 MPa and flexural strength of ~190 MPa, making it suitable for load-bearing applications. Additionally, the PGC-350 material shows small mass loss (~5%) and reasonably low thermal expansion (2.5 × 10^−6^/°C) when heated to 350 °C again. These properties suggest the potential of PGC for high-temperature applications. As a demonstration, PGC formed at 350 °C was employed to fabricate molds to press chalcogenide glass blanks, which exhibited favorable molding results for various surface morphologies.

## 1. Introduction

Carbon-based materials are used extensively in various fields, serving as electrodes in electrochemistry and as molds or linings in high-temperature applications. As an example, graphite is commonly used for high-temperature glass molding [1,2,3] due to its light weight and machinability; however, its low strength leads to frequent mechanical failure. To overcome this problem, ceramic molds [1,4,5] can serve as a replacement as they offer higher strengths and much better mechanical integrity, but the cost is greatly increased because ceramics are brittle and difficult to cut. It is noted that another form of bulk carbon, possessing similar mechanical properties compared to ceramics and similar electric conductivity compared to graphite, known as glassy carbon (GC), is relatively less explored. GC was first discovered in the 1960s by Lewis et al. [6] and further explored by Yamada et al. [7], who investigated the effects of its pyrolysis process at different temperatures. Today, industrialists and researchers use glassy carbon in some niche applications such as electrochemical electrodes [8,9], nanoimprint molds [10,11,12], dental implants [13,14], the detection of toxic substances [15,16], etc. Nevertheless, existing commercial glassy carbon manufacturing processes have a thickness limit of 6 mm [17,18] (fracture occurs otherwise), rendering the production of large-sized components challenging. 

To overcome this manufacturing limitation of glassy carbon, we hypothesize that the fracture of a bulk GC during pyrolysis is caused by the generation of voids, which were expanded and pressurized during the subsequent heating and decomposition after their appearance, finally leading to cracks and disintegration. However, in the heating process, there could be a critical temperature, *T*_C_, below which existing pores caused by precursor curing do not pressurize or expand and the precursor can be sufficiently carbonized to achieve good mechanical properties. In further heating, pores may be pressurized and expanded, leading to cracks in the carbon-based structure. To be more specific, Jenkin et al. [17] reported three stages in the pyrolysis of phenol-formaldehyde (PF) resins to prepare GC, including the pre-carbonization, carbonization, and dehydrogenation stages. Among them, the initial stage (pre-carbonization) involves the carbonization of highly cross-linked precursors, transpiring within a temperature range from room temperature to 400 °C. We thus expect that *T*_C_ could also be below 400 °C. The work of Li et al. [19] demonstrated the fabrication of silicon anodes using partially carbonized polymers as binders with favorable strength and conductivity. Their work suggested that the products of partially carbonized resin may exhibit exceptional mechanical properties. This potential opens avenues for applications requiring light weight, high strength, and inertness at temperatures below *T*_C_. 

In this study, we cured and then pyrolyzed PF resins following the protocol established in [20] to obtain products in different pre-carbonization stages and called them pre-carbonized glassy carbon (PGC). First, the PGC samples obtained at different temperatures were scrutinized to understand the mechanism of cracking. Then, we showed that the PGC products without the formation of large pores or cracks can exhibit exceptional mechanical properties. Note that PGC products can be precisely shaped due to the formability of precursors (by curing liquid resin) and because the PGC can be used at elevated temperatures (below its pyrolysis temperature). We demonstrate the molding application of PGC for shaping chalcogenide glass components used in infrared optics.

## 2. Experimental Section

We first investigated the pyrolysis mechanism based on the TGA (thermogravimetric analysis) results and characterized the mechanical properties and cross-sectional morphology using a universal testing machine (UTM) and scanning electron microscopy (SEM), respectively. Based on the obtained results, several molds were prepared using the proposed fabrication methods, and some chalcogenide glass samples were molded to produce glass components with various surface morphologies.

### 2.1. Sample Preparation

**Figure 1:**
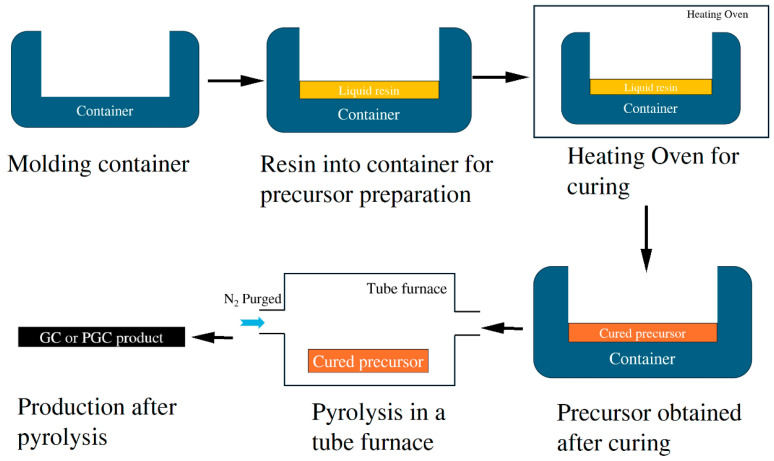
A flowchart of PGC fabrication.

The schematics of sample preparation are illustrated in Figure 1. In this study, the PF resin, sourced from Henan Borun Casting Materials Co., Ltd. (Zhengzhou, China), exhibited a room-temperature viscosity between 16 and 19 Pa·s and a carbon mass content of 40–45%. The resin underwent curing in an incubator at 70–110 °C following degassing in a low-vacuum environment for 1 h. The curing duration was 18–22 h. Subsequently, the cured precursor underwent pyrolysis in a tube furnace under a nitrogen (N2)-purged atmosphere. The pyrolysis process involved heating the GC precursor from room temperature to several temperatures up to 1000 °C at a rate of 1 °C/min, soaking at a maximum temperature for 60 m, and finally cooling in the furnace. After pyrolysis, the sample surfaces were scanned using a surface profiler (Bruker DektakXT Surface Profiler, Billerica, MA, USA) to obtain the profiles and roughness. 

### 2.2. Characterization

#### 2.2.1. Thermal and Morphological Analyses

Thermogravimetric analysis (TGA) and differential thermal analysis (DTA) were conducted in a nitrogen environment using a Rigaku STA8122 thermogravimetric analyzer (Tokyo, Japan). To examine the mass variation during the precursor pyrolysis process, the precursor was subjected to heating from room temperature to 500 °C at a rate of 10 °C/min in a nitrogen environment with a flow rate of 100 mL/min. The further mass changes in PGC during heating were examined under the same conditions. A dilatometer (Cryoall C15V, Beijing, China) was employed to characterize the coefficient of thermal expansion (CTE) exhibited by the PGC samples. Specifically, a sample measuring 4 × 4 × 25 mm^3^ was subjected to controlled heating, ranging from room temperature to 500 °C, at a rate of 10 °C/min. This experimental procedure was conducted under argon protection to ensure an inert environment, facilitating precise measurements of the coefficient of thermal expansion during the molding process.

To investigate the cross-sectional morphologies of samples pyrolyzed at different temperatures, a scanning electron microscope (SEM-TESCAN VEGA3, Brno, Czech Republic) equipped with an energy-dispersive X-ray (EDX, Oxford Instruments, Abingdon, UK) was employed. Some samples were scanned using a 3D micro-CT (XPloreVista 2000 4D, Suzhou, China), with a voltage of 40 kV, a current of 110 μA, a resolution of 5.138 μm, and 1200 scans per sample.

#### 2.2.2. X-Ray Diffraction (XRD)

XRD measurements were conducted using a Rigaku SmartLab 9 kW X-ray diffractometer (Tokyo, Japan) with a rotating anode X-ray tube, operating at a potential of 45 kV and a current of 200 mA. During each experiment, the XRD scan covered a 2θ range from 10° to 80°, with a step size of 0.01° and an integration time of 10 s per point, resulting in a total scan time of approximately 10 min.

#### 2.2.3. Mechanical Tests

**Figure 2:**
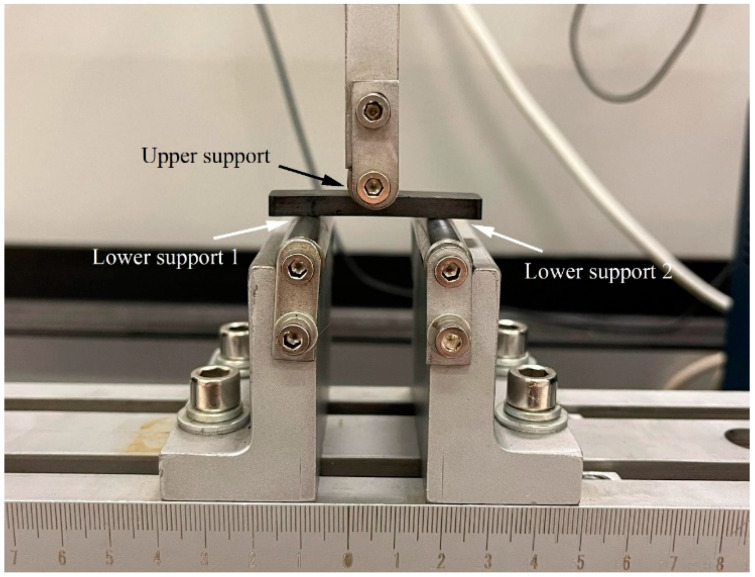
Illustration of three-point bending testing experimental set-up.

The compressive strengths of samples pyrolyzed at different temperatures were measured by using a universal testing machine (GP-TS2000M, Gopoint, Chennai, India). Each sample was placed on a sample stage and pressed at a rate of 2 mm/min until the sample was broken. We also employed the universal testing machine to conduct three-point bending tests with PGC samples with a width of *w* = 10 mm, a height of *h* = 4.42 mm, and a length *l* larger than the distance between two supports of 30 mm, as illustrated in Figure 2. The flexural strength $\sigma_{f}$ can be calculated using the maximum load (F) applied to the specimen, obtained from the following equation:

(1) $$\sigma_{f}=\frac{3Fl}{2wh^{2}}$$

The flexural strain ε can be calculated using the deflection (D) of the middle point of the specimen, obtained from the following equation:

(2) $$\varepsilon =\frac{6Dh}{h^{2}}$$

The hardness of each PGC sample was measured by a Rockwell hardness tester (LC-200RB, Future Tech, Sydney, NSW, Australia). The samples were placed on the sample stage and pressed down with a load of 5 kg to obtain the hardness values.

## 3. Results and Analysis

### 3.1. Characterization of Pyrolysis Induced Cracking 

**Figure 3:**
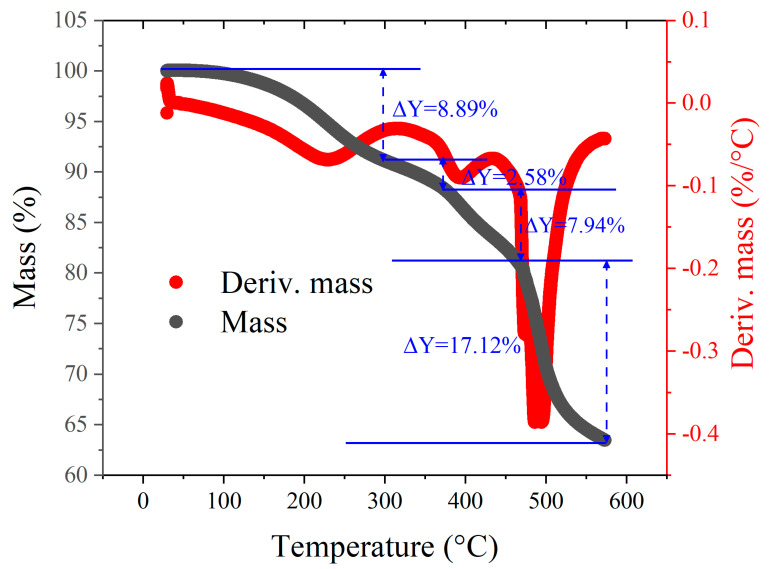
TGA result of a cured PF sample.

Researchers have investigated the thermal decomposition characteristics of PF resins. For example, Chang et al. [21] reported the mass changes in PF resin during heating from 20 to 750 °C at a rate of 5 °C/min in a helium atmosphere. Similarly, Wang et al. [22] presented TGA results for the pyrolysis of PF resin from 90 to 1000 °C in a nitrogen atmosphere. A common observation in their work was rapid mass loss first at around 200 °C and then in a temperature range of 400 to 600 °C. However, considering the variability of PF resins from different manufacturers, we conducted TGA for the specific PF resin used in our study, as illustrated in Figure 3. The mass reduction of 8.89% at 300 °C is primarily due to the escape of water vapor generated by internal reactions, with the rate of mass loss stabilizing at around 300 °C. Beyond this temperature, the sample begins initial carbonization, leading to an increase in the rate of mass loss. At around 370 °C, this rate further accelerates. Between 370 °C and 500 °C, the sample’s mass decreases by 17.12%, as the temperature rise intensifies carbonization, producing gaseous by-products such as carbon dioxide and methane. Based on this point, we called the pyrolytic samples at maximum temperatures below 500 °C pre-carbonized glassy carbon (PGC) and those above 500 °C glassy carbon (GC). Note that for GC samples, non-carbon atoms are not fully driven away, but the mass loss is much gentler during further heating at temperatures above 500 °C. In the following section, we denote the PGC and GC samples using their maximum pyrolysis temperature; for example, PGC-300 means PGC samples pyrolyzed at 300 °C, and it is expected that after some amount of H and O atoms are driven away (mainly in the form of water vapor [23,24]) in the PGC samples, the remaining carbon-rich material may exhibit exceptional mechanical properties suitable for practical applications. 

**Figure 4:**
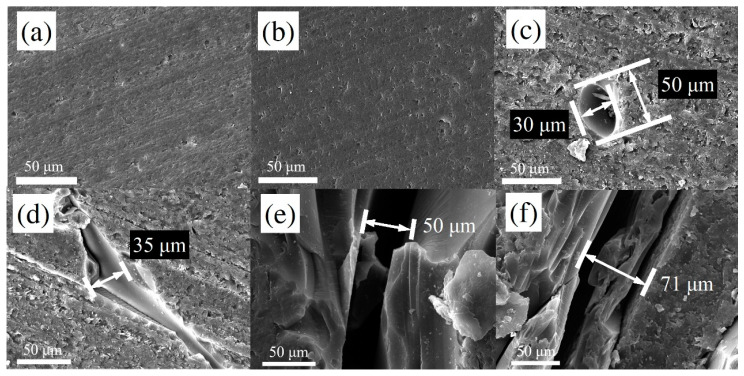
Cross-sectional morphologies of (**a**) PGC-300, (**b**) PGC-350, (**c**) PGC-400, (**d**) PGC-450, (**e**) PGC-600, and (**f**) GC-1000.

To investigate the evolution of internal defects and pores in the products during the pyrolysis of the precursor up to 1000 °C, we performed cross-sectional SEM observations for PGC and GC samples obtained between 300 and 1000 °C. The PGC and GC samples were first mounted in epoxy and then sectioned using a diamond cutter (Isomet 5000, Buehler, Sydney, NSW, Australia) for observation. The typical results are shown in Figure 4, which are from the PGC samples obtained at 300, 350, 400, and 450 °C and the GC samples obtained at 600 and 1000 °C.

Figure 4a,b exhibit dense cross-sectional morphologies for the PGC samples obtained at 300 and 350 °C, respectively. One can observe small voids (below 5 μm), and there are no large pores or apparent cracks. However, further heating to 400 °C led to large elliptical pores with major and minor axes of approximately 30 and 50 μm, respectively, as shown in Figure 4c, caused by the expediated gas generation and during pyrolysis at 400 °C. Figure 4d–f demonstrate cracks caused by further heating. Figure 4d shows a crack with a maximum width of 35 μm in the cross-section of a PGC-450 sample. It is noteworthy that there is a pore (in the upper right corner of the image) connecting to the crack, leading to speculation that this crack originated from the pore, which was pressurized due to the accumulation of gas products inside this pore. In Figure 4e, a crack with a width of 50 μm was observed in the cross-section of a PGC-600 sample, and in Figure 4f, a crack with a width of 71 μm was observed on the cross-section of a GC-1000 sample. These micrographs indicate that with the increase in pyrolysis temperature, the internal large pores are pressurized, leading to the opening of cracks, and that the pore generation and cracking processes likely start from a temperature around 400 °C. 

**Figure 5:**
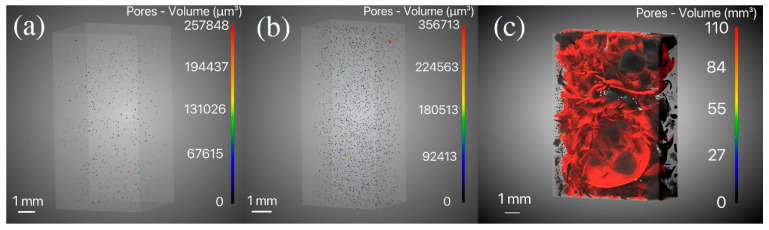
Micro-CT results of (**a**) PGC-350, (**b**) PGC-400, and (**c**) GC-1000.

We conducted future micro-CT scanning for the PGC and GC samples obtained at 350, 400, and 1000 °C, respectively. Figure 5a displays a 3D image of the PGC-350 bulk sample, with dimensions of 10 × 5 × 3 mm^3^. From the image, it can be observed that the sample contains uniformly distributed small cavities. In the figure, the colors indicate pore volumes determined by the software associated with the micro-CT (Avizo 2022.2), and the largest one (i.e., the red one) has a volume of 257,848 μm^3^, equivalent to a spherical cavity with a radius of 39.48 μm. It should be noted that such large pores (i.e., tens of microns in radius) are very rare in the PGC-350 sample. Most of the pores are blue or black colored with radii well below 10 μm. Hence, it is speculated that the small number of large pores was formed in the precursor curing process instead of pyrolysis. Figure 5b illustrates the micro-CT result of a PGC-400 bulk sample with dimensions of 11 × 5 × 5 mm^3^. Apparently, the number of internal pores significantly increases compared to that of PGC-350, and the radius of the largest pore becomes 45 μm. It should be noted that the pores in the PGC-400 sample remain isolated cavities, i.e., cracks are not formed. This is consistent with the result shown in Figure 4c. The SEM and micro-CT results of PGC-350 and PGC-400 samples indicate pore formation and expansion due to the release of gaseous products. However, these pores do not lead to cracks, probably because the gaseous products can still diffuse to free surfaces without causing a buildup of internal pore pressure, and Figure 4d–f indicate that further heating to higher temperatures leads to cracks because it becomes more difficult for gasses to escape. As a comparison, the micro-CT result of GC-1000, with dimensions of 14 × 5 × 3.5 mm^3^, is shown in Figure 5c, wherein a completely different morphology of internal cavities is demonstrated. Although the GC-1000 sample was not fractured, its interior contains many interconnected cracks forming a network of cavities, and such a cavity network can have a very large volume of ~110 mm^3^. An interesting phenomenon associated with the GC-1000 sample is that the cracks do not reach the surfaces. They remain inside the bulk material without causing fracture (fracture does happen when the sample thickness is larger). This indicates that the free surfaces of a bulk GC sample can be free from cracks because gas can escape and there is no pressure buildup. 

### 3.2. Numerical Model of Pyrolysis-Induced Cracking 

We provide a numerical model to better substantiate our hypothesis regarding the causes of cracks during the pyrolysis process. This model is based on the phase-field approach [25]. We define an arbitrary elastic geometric body to describe a bulk precursor $\Omega \subset R^{d}$ (where *d* represents the dimensionality of the space) with the boundary ∂Ω, and the interior of this geometric body contains discrete, non-continuous cracks denoted by Γ. 

Consider the Griffith fracture energy *G*_C_, body forces b, traction forces t, and elastic energy $\psi_{e}$. The total mechanical energy contributes to the overall potential energy of the system $\psi_{tot}$, given by

(3) $$\psi_{tot}=\int_{\Omega }\psi_{e}\left(\varepsilon^{e}\left(u\right),\Gamma \right)dV+\int_{\Gamma }G_{c}dA-\int_{\Omega }b\cdot udV-\int_{\partial \Omega }t\cdot udA$$

where $\varepsilon^{e}$, u, and $G_{c}$ are the elastic strain tensor, displacement vector, and critical fracture energy density, respectively. The system is described using a phase-field model with a variable ϕ∈[0, 1], with *ϕ* = 1 corresponding to the cracked regions, *ϕ* = 0 the intact regions, and ϕ∈(0, 1) the diffusive interfaces between cracked region and the surrounding intact region. Miehe et al. [26] provide an expression for the fracture energy density induced by extended cracks, expressed as follows:

(4) $$\gamma \left(\phi ,\nabla \phi \right)=\frac{1}{2l_{0}}\phi^{2}+\frac{l_{0}}{2}{\left|\nabla \phi \right|}^{2}$$

where *l*_0_ is a parameter controlling the diffusive interface thickness. Consequently, the energy release due to fracture is approximated as follows [27]:

(5) $$\int_{\Gamma }G_{c}dA\approx \int_{\Omega }G_{c}\gamma \left(\phi ,\nabla \phi \right)dV$$

It is hypothesized that tension is the only factor to cause cracking. As a result, the elastic strain energy can be decomposed into tensile and compressive components, following the approach suggested in ref. [28]. To achieve this decomposition, the elastic strain tensor ***ε****^e^* is analyzed as follows:

(6) $$\left\{\begin{array}{l} \varepsilon_{+}^{e}=\sum_{a=1}^{d}{\left\langle \varepsilon_{a}^{e}\right\rangle }_{+}n_{a}⊗n_{a}\\ \varepsilon_{-}^{e}=\sum_{a=1}^{d}{\left\langle \varepsilon_{a}^{e}\right\rangle }_{-}n_{a}⊗n_{a} \end{array}\right.$$

where $\varepsilon_{+}^{e}$ and $\varepsilon_{-}^{e}$ represent the tensile and compressive elastic strain tensors, respectively, and $\varepsilon_{a}^{e}$ and $n_{a}$ denote the principal elastic strain and its corresponding direction vector, respectively. The operators ${\left\langle \cdot \right\rangle }_{+}$ and ${\left\langle \cdot \right\rangle }_{-}$ are defined as ${\left\langle \cdot \right\rangle }_{+}=\frac{\cdot +\left|\cdot \right|}{2}$ and ${\left\langle \cdot \right\rangle }_{-}=\frac{\cdot -\left|\cdot \right|}{2}$. 

Based on Equation (6), the strain energy density can be resolved into two parts:

(7) $$\psi_{e}\left(\varepsilon^{e}\left(u\right)\right)=\psi_{e}^{+}\left(\varepsilon^{e}\left(u\right)\right)+\psi_{e}^{-}\left(\varepsilon^{e}\left(u\right)\right)$$

with

(8) $$\left\{\begin{array}{l} \psi_{e}^{+}=\frac{1}{2}\lambda_{0}{\left\langle \mathrm{tr}\left(\varepsilon^{e}\right)\right\rangle }_{+}^{2}+\mu_{0}tr\left(\varepsilon_{+}^{e}\cdot \varepsilon_{+}^{e}\right)\\ \psi_{e}^{-}=\frac{1}{2}\lambda_{0}{\left\langle \mathrm{tr}\left(\varepsilon^{e}\right)\right\rangle }_{-}^{2}+\mu_{0}tr\left(\varepsilon_{-}^{e}\cdot \varepsilon_{-}^{e}\right) \end{array}\right.$$

where *λ*_0_ and *μ*_0_ represent Lamé constants.

In cracked regions, stiffness is reduced with *ϕ*. This phenomenon is described by a so-called degradation function *g*(*ϕ*), given by [29]:

(9) $$g\left(\phi \right)=\left(1-k\right){\left(1-\phi \right)}^{2}+k$$

where *k* is chosen to be sufficiently small and positive to ensure the stability of the numerical scheme. For this study, *k* is set to 1 × 10^−5^.

With the procedures above, the strain energy density can be delineated as follows:

(10) $$\psi_{e}\left(\varepsilon^{e}\left(u\right),\phi \right)=g\left(\phi \right)\psi_{e}^{+}\left(\varepsilon^{e}\left(u\right)\right)+\psi_{e}^{-}\left(\varepsilon^{e}\left(u\right)\right)$$

With Equations (3), (5), and (10), the total Lagrangian energy functional can be formulated as follows:

(11) $$\begin{array}{l} L=\frac{1}{2}\int_{\Omega }\rho \dot{u}:\dot{u}dV-\int_{\Omega }G_{c}\gamma \left(\phi ,\nabla \phi \right)dV-\int_{\Omega }g\left(\phi \right)\psi_{e}^{+}\left(\varepsilon^{e}\left(u\right)\right)+\psi_{e}^{+}\left(\varepsilon^{e}\left(u\right)\right)dV\\ +\int_{\Omega }b\cdot udV+\int_{\partial \Omega }t\cdot udA \end{array}$$

where *ρ* is the density of GC.

By setting the functional derivatives of *L* with respect to **u** and setting *ϕ* to zero (thus minimizing the Lagrange value), the corresponding equations of motion are derived as follows:

(12) $$\left\{\begin{array}{l} \nabla \sigma +b=\rho \ddot{u}\;\mathrm{in}\;\Omega \\ \left[\frac{2l_{0}\left(1-k\right)\psi_{e}^{+}}{G_{c}}+1\right]\phi -l_{0}^{2}\nabla^{2}\phi =\frac{2l_{0}\left(1-k\right)\psi_{e}^{+}}{G_{c}}\;\mathrm{in}\;\Omega \\ \sigma \cdot n=t\;\mathrm{on}\;\partial \Omega \\ \nabla \phi \cdot n=0\;\mathrm{on}\;\partial \Omega \end{array}\right.$$

where $\sigma =g\left(\phi \right)\frac{\partial \psi_{e}^{+}}{\partial \varepsilon^{e}}+\frac{\partial \psi_{e}^{-}}{\partial \varepsilon^{e}}$, and **n** represents the outward normal vector on the boundaries, ∂Ω.

To prevent self-healing of the crack, an irreversible condition is imperative. Consequently, we define a historical strain field *H*, which is the maximum of the strain energy $\psi_{e}^{+}$ [30,31], given as follows:

(13) $$H\left(x,t\right)=\underset{s\in [0,t]}{max}\left\{\psi_{e}^{+}\left(x,s\right)\right\}$$

Replacing $\psi_{e}^{+}$ with *H* in Equation (12) yields

(14) $$\left\{\begin{array}{l} \nabla \sigma +b=\rho \ddot{u}\;\mathrm{in}\;\Omega \\ \left[\frac{2l_{0}\left(1-k\right)H}{G_{c}}+1\right]\phi -l_{0}^{2}\nabla^{2}\phi =\frac{2l_{0}\left(1-k\right)H}{G_{c}}\;\mathrm{in}\;\Omega \\ \sigma \cdot n=t\;\mathrm{on}\;\partial \Omega \\ \nabla \phi \cdot n=0\;\mathrm{on}\;\partial \Omega \end{array}\right.$$

With Equation (6), the stress tensor **σ** can be expressed as follows:

(15) $$\sigma =\frac{\partial \psi_{e}}{\partial \varepsilon^{e}}=\left[\left(1-k\right)\left(1-\phi^{2}\right)+k\right]\left[\lambda_{0}{\left\langle \mathrm{tr}\left(\varepsilon \right)\right\rangle }_{+}I+2\mu_{0}\varepsilon_{+}^{e}\right]+\lambda_{0}{\left\langle \mathrm{tr}\left(\varepsilon \right)\right\rangle }_{-}I+2\mu_{0}\varepsilon_{-}^{e}$$

and the stiffness matrix (fourth-order tensor) D is obtained as below [32,33]:

(16) $$\begin{array}{r} D=\frac{\partial \sigma }{\partial \varepsilon^{e}}=\lambda_{0}\left\{\left[\left(1-k\right)\left(1-\phi^{2}\right)+k\right]H_{\varepsilon }\left\langle \mathrm{tr}\left(\varepsilon \right)\right\rangle +H_{\varepsilon }\left\langle -\mathrm{tr}\left(\varepsilon \right)\right\rangle \right\}J\\ +2\mu_{0}\left[\left(1-k\right)\left(1-\phi^{2}\right)+k\right]\frac{\partial \varepsilon_{+}}{\partial \varepsilon }+2\mu_{0}\frac{\partial \varepsilon_{-}}{\partial \varepsilon } \end{array}$$

where $H_{\varepsilon }\left\langle \cdot \right\rangle$ is the Heaviside function, defined as $H_{\varepsilon }\left\langle x\right\rangle =1$ when x>0 and $H_{\varepsilon }\left\langle x\right\rangle =0$ when x≤0. To ensure the positive definiteness of D, we adopt the modification of the principal strains as described in ref. [34]:

(17) $$\left\{\begin{array}{l} \varepsilon_{1}=\varepsilon_{1}\left(1+\delta \right)\;\mathrm{if}\;\varepsilon_{1}=\varepsilon_{2}\\ \varepsilon_{3}=\varepsilon_{3}\left(1-\delta \right)\;\mathrm{if}\;\varepsilon_{2}=\varepsilon_{3} \end{array}\right.$$

with *δ* = 1 × 10^−9^ in this study.

For cases involving initial cracks Γ and to prevent their self-healing, the initial magnitude of the strain history field *H*_0_(***x***) = *H*(***x***, 0) can be predefined as follows:

(18) $$H_{0}\left(x\right)=B\left\{\begin{array}{ll} \frac{G_{c}}{4l_{0}}\left(1-\frac{d\left(x,\Gamma \right)}{l_{0}}\right) & d\left(x,\Gamma \right)\leq l_{0}\\ 0 & d\left(x,\Gamma \right)>l_{0} \end{array}\right.$$

where *d*(***x***, *Γ*) represents the closest distance from point ***x*** to the discontinuity *Γ*, and *B* is a scalar [30] from 0 to 1000, resulting in *H*_0_(***x***) varying from 0 to 1.

To address this initial-boundary-value problem, Equations (13), (14), and (16) are implemented in a finite-element solver, COMSOL Multiphysics 6.2. The parameters employed in the numerical simulation are detailed in Table 1. The simulation codes can be made available upon request.

**Figure 6:**
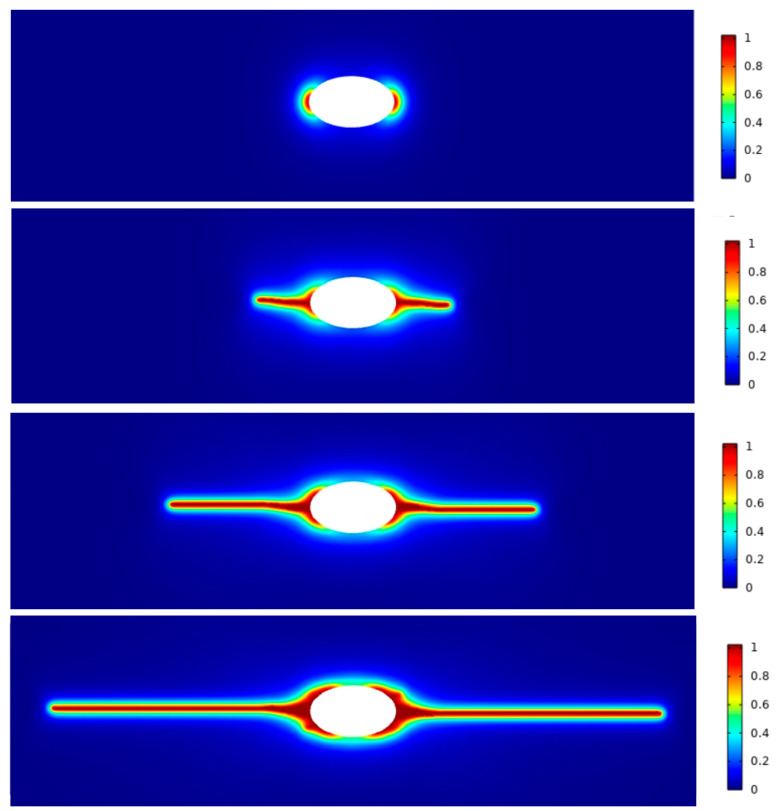
Simulation of crack initiation and propagation when an elliptical hole is pressurized.

**Figure 7:**
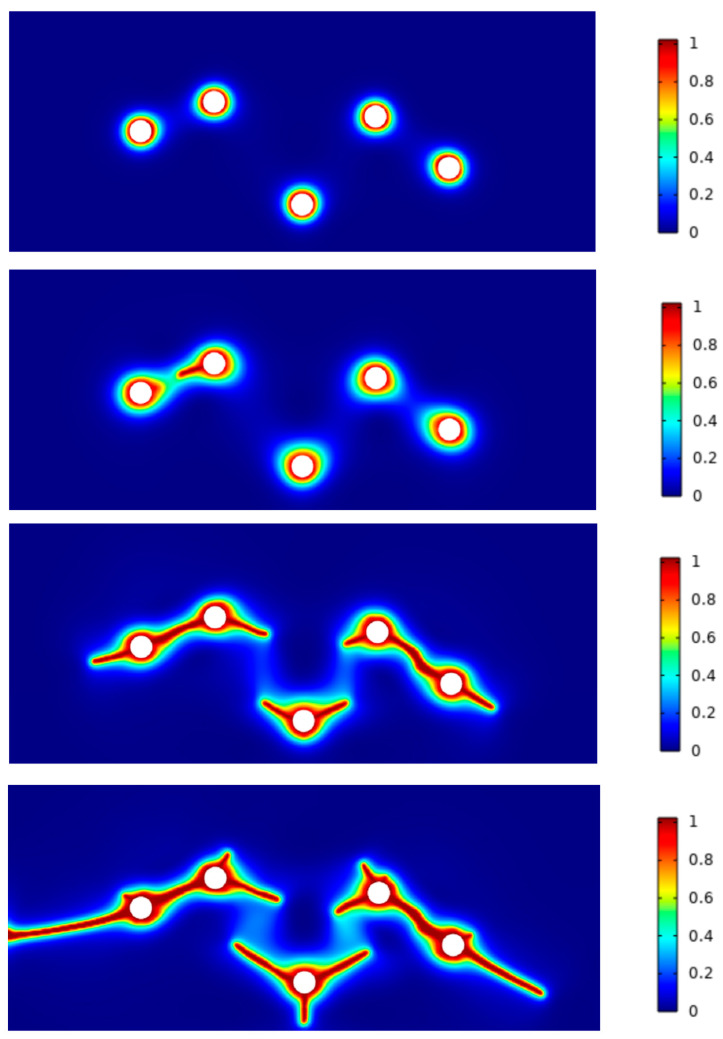
Crack propagation and connection when multiple holes were pressurized.

For GC cracking, we conduct two-dimensional (2D) simulations for a case where a pressurized elliptical cavity leads to crack propagation as shown in Figure 6. The elliptical hole has a major axis of 50 μm and a minor axis of 30 μm located at the center of the section. During pyrolysis, a GC structure forms on the exterior of the precursor, while gaseous products diffuse into the cavity, causing an increase in pore pressure. When the pore pressure increases, causing tensile stress at the pore edges larger than the critical stress *σ_cr_*, cracks originate at the ends of the major axis of the elliptical cavity and extend. In our experiments, such lateral cracks and separation of GC samples were often met when thick precursors were pyrolyzed into GC samples (e.g., see Figure 4d). We also simulated cases of multiple circular holes, as shown in Figure 7. These holes led to connected cracks inside a bulk GC sample, which is analogous to Figure 5c. Note that the computational model only rationalizes the hypothesized process of cracking. The parameters used in the model (Table 1) are not calibrated against experimental results, and the numerical examples are only 2D due to the limitation in computational resources (3D cases would be extremely demanding for phase-field modeling). Hence, in this work, we used these numerical examples to visual the process of cracking and provide an analogy to the experimental observations. In the numerical model, we assumed a gradual increase in pore pressure. This increase can be replaced by a reaction and diffusion process of gaseous products, which can be detailed in future work. 

### 3.3. Properties of Pre-Carbonized Glassy Carbon (PGC)

**Figure 8:**
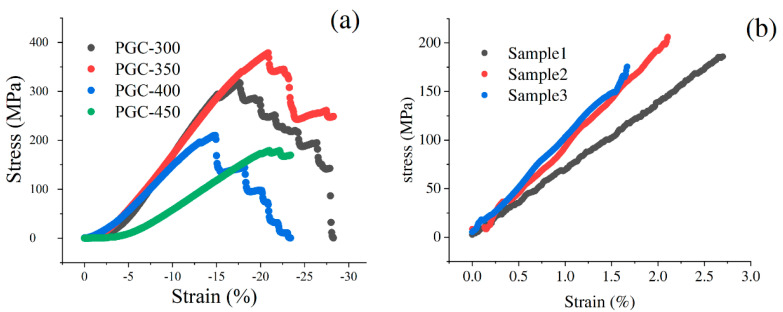
(**a**) Compressive stress–strain curves of PGC samples; (**b**) flexural stress–strain curves of PGC-350 samples.

Because the bulk GC samples were generally severely cracked internally, we turned to study properties of PGC samples, which are less cracked and also less investigated in the literature. Compression tests were performed for four types of PGC samples, and the results are exhibited in Figure 8a. Notably, fracture occurs at a compressive strain in the range of 15–21% for all four samples. Among them, PGC-350 exhibits the highest compressive stress, attaining approximately 370 MPa. With a density of ~1.4g/cm^3^, the specific compressive strength is ~260 Nm/g, which is similar to the properties of maraging steel and titanium alloy. The compressive strength decreases sequentially for PGC-300, PGC-400, and PGC-450. For PGC-400 and PGC-450, it is clear that the decrease in compressive strength is due to internal cracks, as shown in Figure 4c,d. For PGC-300, its lower compressive strength compared to PGC-350 is caused by insufficient carbonization. The TGA results, as shown in Figure 3, indicate that the mass change from 300 to 350 °C is approximately 5%. The release of volatile compounds and mass reduction from 300 to 350 °C imply an increased degree of carbonization, leading to a denser structure and improved compressive strength. Between 300 and 350 °C, gaseous products like methane and carbon monoxide continuously form, and some accumulate within initial pores, leading to a rise in internal pore pressure. However, they do not cause severe porosity or cracking; therefore, the PGC samples exhibit the trend whereby their mechanical strength increases with temperature.

In the literature, reactions from 300 to 450 °C have been described. From 300 to 400 °C, the predominant reactions involve the cleavage of terminal benzene rings, resulting in the formation of phenol and cresol [39,40]. Concurrently, hydroxyl functional groups undergo condensation reactions with methyl groups, leading to a minor decrease in mass due to the generation of vapor. Simultaneously, the recombination of C-C bonds induces structural evolution, contributing to an increase in compressive strength. These reactions promote structural reorganization, allowing carbon atoms to bond more tightly, thereby enhancing the overall carbonization of the material. This process results in a more stable structure, free from large pores or significant cracks, as confirmed by the SEM and TGA analyses demonstrated in Figure 3 and Figure 4. Beyond 400 °C, intermolecular etherification reactions occur among hydroxyl functional groups, producing more vapor, while gaseous products react with methyl groups to generate hydrogen, methane, and carbon monoxide. The escape of gaseous products and internal reactions contribute to intensified mass loss, accompanied by the formation of pores and cracks within the material. The EDX results of the four samples, as shown in Table 2, evidence that the carbon-to-oxygen atomic ratios for PGC-300 and PGC-350 are nearly identical, while they increase in PGC-400 and PGC-450. The proportion of carbon atoms increases with temperature, indicating that the internal chemical reactions lead to the consumption of oxygen atoms, forming H_2_O and increasing internal pore pressure. Therefore, the cross-sections of PGC-400 and PGC-450 exhibit notable pores and cracks.

**Figure 9:**
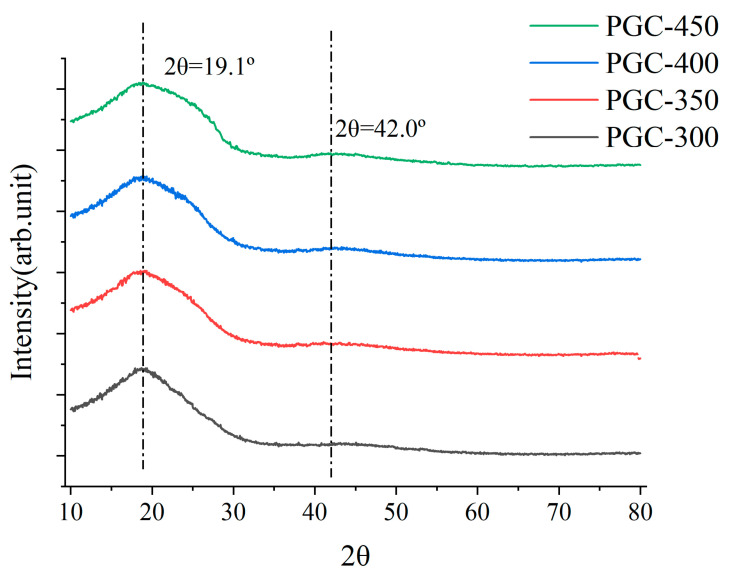
XRD results of PGC samples.

Additionally, we conducted XRD characterization on the PGC samples, and the results are shown in Figure 9. All four PGC samples exhibit two characteristic peaks at 2θ = 19.1° and 2θ = 42.0°, attributed to adjacent chains of linear polymers [41] within the structure. As the pyrolysis temperature increases, the peak width at 2θ = 19.1° broadens noticeably, indicating a higher degree of carbonization and a corresponding increase in the amorphous nature of the internal microstructure with rising temperature.

The hardness of the samples, as shown in Table 2, does not increase with the degree of carbonization. PGC-350 and PGC-400 exhibit relatively higher hardness values of 116.58 and 121.42 HB, respectively. PGC-300 shows the lowest hardness of 94 HB. Interestingly, the hardness of PGC-450 is 107.43 HB, lower than that of PGC-400, which can be attributed to internal cracks. With its high compressive strength and hardness, we expect that PGC-350 is the most promising candidate for load-bearing applications at temperatures below 350 °C, such as mold materials. 

PGC-350 could also be used for structural applications when light weight and high strength are required. For such applications, we conducted three-point bending for PGC-350 samples to determine their flexural strength. The calculated flexural strengths obtained from three samples, as shown in Figure 8b, are 176 MPa, 186 MPa, and 207 MPa, respectively. From these results, it can be inferred that the flexural strength of PGC-350 is ~190 MPa (specific flexural strength ~135 Nm/g) with a tolerance of 9%. In the three-point bending experiment, the flexural modulus of Sample 1 is 7 GPa, while Sample 2 and Sample 3 exhibit a flexural modulus of 10 GPa. This variation is because Sample 1 was produced from an earlier batch. These results indicate the variation in PF resins purchased in different batches. We found that with prolonged storage time, the viscosity of the PF resin gradually increases, and that the increase in resin viscosity may have led to a lower degree of degassing before pre-carbonization in Sample 1. In such a scenario, we would anticipate lower mechanical properties. Given the favorable flexural strength of PGC-350, we expect that it can be applied in the structures to make them strong and lightweight.

**Figure 10:**
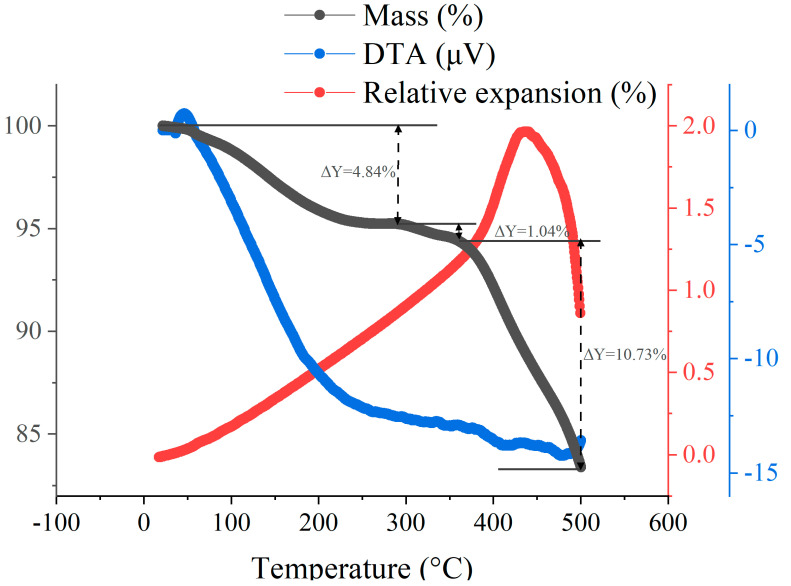
TGA, DTA, and dilatometry results of PGC-350.

To explore the potential of using PGC-350 as a mold material, we conducted TGA, DTA, and CTE tests under the heating process described above, and the results are illustrated in Figure 10. The TGA and DTA results, as shown in Figure 10, reveal distinct stages of thermal decomposition in the PGC sample from room temperature to 500 °C. Initially, from room temperature to 300 °C, the TGA curve shows gradual mass loss of approximately 4.84%. The DTA curve indicates endothermic reactions within this range, suggesting that gasses (mainly H_2_O [17]) are generated and escape, causing mass reduction. As the temperature nears 300 °C, both the mass loss rate and endothermic activities become mild. Between 300 and 370 °C, the TGA curve shows slow mass loss, while the DTA curve exhibits minor endothermic and exothermic oscillations. The exothermic reactions likely correspond to carbon structure formation, while the endothermic signals may indicate decomposition of the sample [42]. From 370 to 500 °C, the rate of mass loss increases significantly (the total mass reduction is 10.73%). During this stage, the DTA curve continues to oscillate but trends toward exothermic behavior at around 470–500 °C. This suggests that gas-producing endothermic reactions persist, while C-C bond formation and progressive carbonization intensify. Concurrently, the dilatometry results in Figure 10 indicate that the length of the PGC sample increases approximately linearly in the range from room temperature to 350 °C, leading to a CTE of about 2.5 × 10^−5^/°C, then more steeply from 350 to 450 °C, and finally decreases between 450 and 500 °C due to the substantial production and escape of methane and carbon dioxide in this temperature range [39]. As the dilatometry and TGA results suggest minimal mass loss and a relatively low CTE below 350 °C, we expect that PGC-350 is a suitable mold material for infrared glass molding, with typical molding temperatures below 300 °C.

### 3.4. Example of Application of PGC 

**Figure 11:**
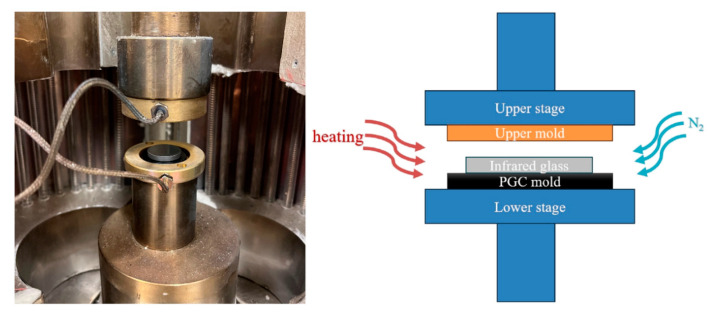
Photo and schematics of glass-molding system.

To exemplify a possible application of PGC requiring good mechanical strength and sufficient chemical inertness at elevated temperatures, we choose to use PGC as a mold for precision molding. We conducted molding experiments using a computer-controlled glass pressing machine (WDW-10, Xinshijinm Jinan, China). The equipment and schematics are illustrated in Figure 11. The load cell of the machine was 10 kN with a tolerance of ±0.5%, and the molding process was displacement control with a maximum actuator stroke of 200 mm and accuracy of 0.01 mm. The heating chamber employed far-infrared tube heaters with a total power of 3 kW. In the molding experiments, a PGC mold was used as the lower mold, and a graphite plate was used as the upper mold. The system was heated from room temperature to 300 °C at a rate of 10 °C/min under a nitrogen atmosphere. After reaching 300 °C, the sample was maintained at this temperature, followed by molding under a force of 300 N for 200 s. Subsequently, the molded infrared glass was naturally cooled and removed from the lower mold.

**Figure 12:**
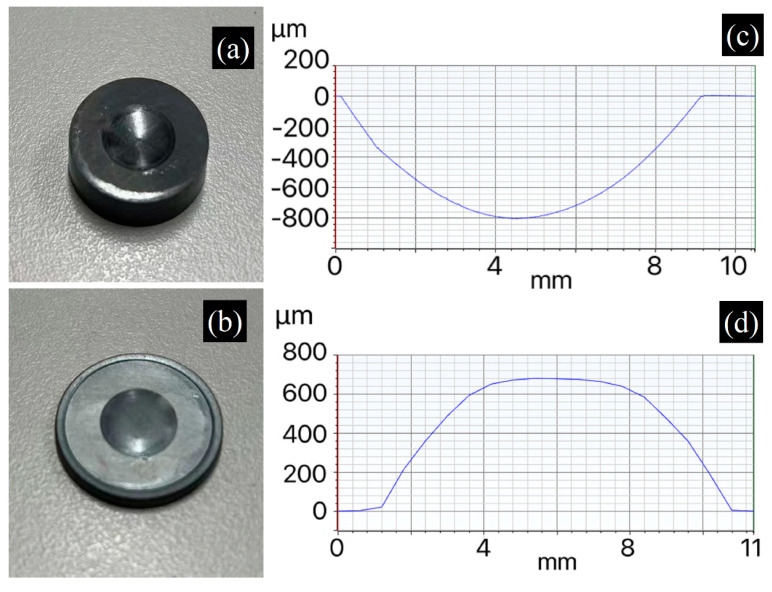
(**a**) PGC mold with concave surface morphology and (**b**) its molded Ge_28_Se_60_Sb_12_ glass. Line scan results of surface morphologies of (**c**) PGC mold and (**d**) molded glass.

As PGC-350 exhibits the best thermomechanical performance, it was selected as the mold material for infrared glass molding. Molds with concave or convex surface profiles were fabricated to press 3 mm thick infrared glass (Ge_28_Se_60_Sb_12_) blanks. Figure 12a,b exhibits a concave PGC-350 mold and the resulting molded glass, respectively. The surface morphology was measured, and the line scan results are presented in Figure 12c,d, demonstrating an effective molding outcome. The maximum depth of the concave mold profile was 800 μm, and the feature height of the molded glass was 680 μm, resulting in a fill ratio of approximately 85%. This incomplete filling may be attributed to the large depth of the mold surface cavity and insufficient fluidity of the heated glass. However, this result demonstrates that PGC can be an excellent mold material for infrared glass molding considering the low processing cost to achieve various surface profiles (i.e., without the need to machine hard materials).

**Figure 13:**
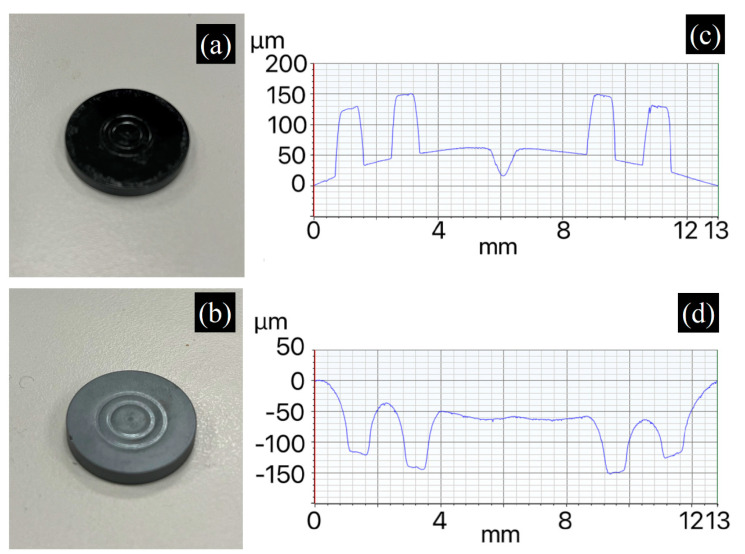
(**a**) PGC mold with annular surface morphology and (**b**) its molded Ge_28_Se_60_Sb_12_ glass. Line scan results of surface morphologies of (**c**) PGC mold and (**d**) molded glass.

**Figure 14:**
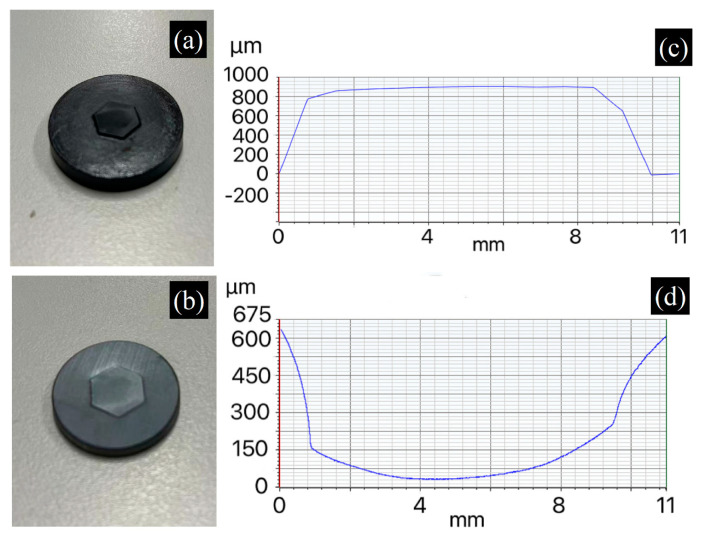
(**a**) PGC mold with hexagonal surface morphology and (**b**) its molded Ge_28_Se_60_Sb_12_ glass. Line scan results of surface morphologies of (**c**) PGC mold and (**d**) molded glass.

We also fabricated PGC molds with different surface morphologies for infrared glass-molding experiments. Figure 13 and Figure 14 depict molds with annular and hexagonal surface morphologies, respectively. Figure 13a,b show the annular mold morphologies and the resulting molded infrared glass, respectively. The morphologies (line scans) of the mold and glass are shown in Figure 13c,d, respectively. The height of the mold’s annular features and the depth of the annular grooves in the molded glass are well matched at 150 μm, demonstrating good replication. Figure 14a,b display hexagonal mold morphologies and resulting molded infrared glass, respectively. Surface line scans of these samples are shown in Figure 14c,d, respectively. The height of the mold’s hexagonal protrusion and the depth of the hexagonal pocket in the molded glass are 900 μm and 600 μm, respectively. In this case, the sharp corners of the hexagon make glass deformation more difficult, leading to a lower ratio of replication (66.7%), which can be improved by increasing the molding time or force. 

## 4. Conclusions

This study on pre-carbonized glassy carbon (PGC) elucidated a critical temperature threshold below which pores and cracks do not form, ensuring mechanical integrity in PGC samples. It is noted that prior to pyrolyzing the samples at 350 °C, there are no large pores present internally, and cracks form in the samples treated at 400 and 450 °C. These cracks alter gas transport, transitioning from purely diffusive transport to molar flux. Further, the diffusive pathways are shortened due to crack-induced damage during decomposition. Therefore, the processing temperatures of pre-carbonization thermoset precursors can be determined using TGA and CTE tests alone.

The PGC samples pyrolyzed at 350 °C exhibit a dense microstructure with minimal internal pores, contributing to their high compressive and flexural strengths. The Ge_28_Se_60_Sb_12_ glass-molding experiments conducted using PGC-350 molds showcase good surface replication results for various surface features with submillimeter heights or depths. Additionally, the fabrication of PGC molds involves only curing and partial pyrolysis at 350 °C. Considering this fabrication route, the surface profiles of PGC molds can be achieved in the resin curing stage without much effort in precision machining, implying a low mold manufacturing cost in larger-scale productions, which suggests that PGC molds are a cost-effective method of transferring surface features to a Ge_28_Se_60_Sb_12_ glass surface. Further it is demonstrated that PGC-350 has excellent thermomechanical properties, which would allow it to serve as a lightweight and high-strength structural component.

## Figures and Tables

**Table 1:** Parameters used in the simulation.

*E*, Young’s modulus	21 GPa [35]
*ν*, Poisson’s ratio	0.3 [36]
*G*_c_, critical energy release rate	39.433 J/m^2^
*l*_0_, crack width	5 μm
*ρ*, density	1.45 g/cm^3^ [37]
*σ_cr_*, critical stress	0.91 MPa [38]

**Table 2:** Carbon and oxygen ratios and hardness of PGC samples.

Samples	PGC-300	PGC-350	PGC-400	PGC-450
C (Atomic%)	84.54	84.67	85.71	86.51
O (Atomic%)	15.46	15.33	14.29	13.49
Hardness (HRB)	94	116.58	121.42	107.43

## Data Availability

The original contributions presented in the study are included in the article, further inquiries can be directed to the corresponding author.
